# Atomic Force Microscopy to Elicit Conformational Transitions of Ferredoxin-Dependent Flavin Thioredoxin Reductases

**DOI:** 10.3390/antiox10091437

**Published:** 2021-09-09

**Authors:** Carlos Marcuello, Gifty Animwaa Frempong, Mónica Balsera, Milagros Medina, Anabel Lostao

**Affiliations:** 1 Instituto de Nanociencia y Materiales de Aragón (INMA), CSIC-Universidad de Zaragoza, 50009 Zaragoza, Spain; cma210786@gmail.com (C.M.); giftyfrempong20@gmail.com (G.A.F.); 2 Laboratorio de Microscopías Avanzadas (LMA), Universidad de Zaragoza, 50018 Zaragoza, Spain; 3 Department of Abiotic Stress, Instituto de Recursos Naturales y Agrobiología de Salamanca (IRNASA-CSIC), 37008 Salamanca, Spain; monica.balsera@csic.es; 4 Departamento de Bioquímica y Biología Molecular y Celular, Facultad de Ciencias, Instituto de Biocomputación y Física de Sistemas Complejos (GBsC-CSIC Joint Unit), Universidad de Zaragoza, 50018 Zaragoza, Spain; 5 Fundación ARAID, 50018 Zaragoza, Spain

**Keywords:** thioredoxin reductase, atomic force microscopy, protein interactions, redox-active disulfide, single-molecule methods, homodimers, flavoproteins

## Abstract

Flavin and redox-active disulfide domains of ferredoxin-dependent flavin thioredoxin reductase (FFTR) homodimers should pivot between flavin-oxidizing (FO) and flavin-reducing (FR) conformations during catalysis, but only FR conformations have been detected by X-ray diffraction and scattering techniques. Atomic force microscopy (AFM) is a single-molecule technique that allows the observation of individual biomolecules with sub-nm resolution in near-native conditions in real-time, providing sampling of molecular properties distributions and identification of existing subpopulations. Here, we show that AFM is suitable to evaluate FR and FO conformations. In agreement with imaging under oxidizing condition, only FR conformations are observed for *Gloeobacter violaceus* FFTR (GvFFTR) and isoform 2 of *Clostridium acetobutylicum* FFTR (CaFFTR2). Nonetheless, different relative dispositions of the redox-active disulfide and FAD-binding domains are detected for FR homodimers, indicating a dynamic disposition of disulfide domains regarding the central protein core in solution. This study also shows that AFM can detect morphological changes upon the interaction of FFTRs with their protein partners. In conclusion, this study paves way for using AFM to provide complementary insight into the FFTR catalytic cycle at pseudo-physiological conditions. However, future approaches for imaging of FO conformations will require technical developments with the capability of maintaining the FAD-reduced state within the protein during AFM scanning.

## 1. Introduction

**Figure 1:**
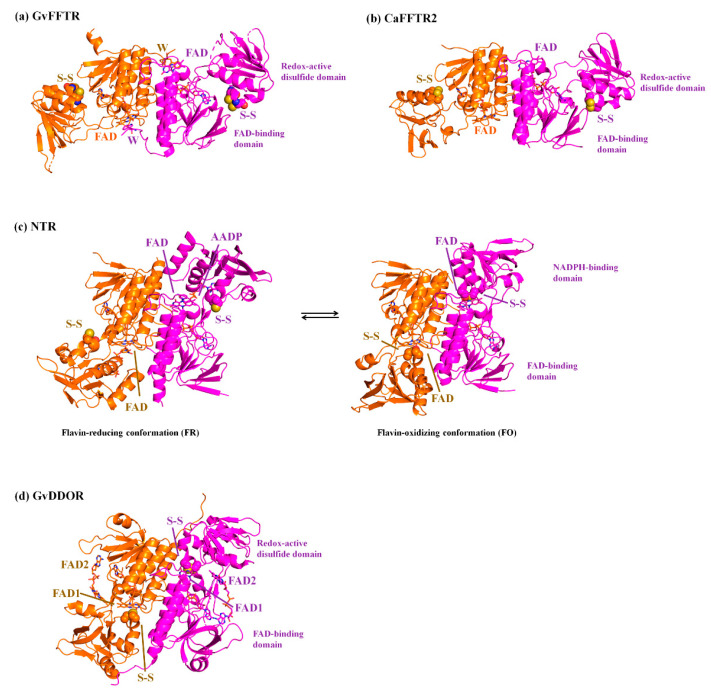
Ribbon representation of the homodimeric protein structures solved by X-ray crystallography of (**a**) GvFFTR; (**b**) CaFFTR2; (**c**) FR and FO conformations of representative bacterial NTRs (PDB codes 1F6M and 1TRB, respectively); and (**d**) GvDDOR. Each protomer in the homodimer is colored in magenta and orange, respectively, and the two domains for a monomer are labeled. FAD cofactors and tryptophan (W) at the C-tail are depicted in stick representation, whereas the Cys amino acids forming part of the redox-active CxxC motif are represented in spheres. Note that for bacterial FR and FO NTR conformations, the CxxC motif has been mutated to SxxC and CxxS, respectively [10,11]. The FR conformation in (–**c**) has been solved in the presence of 3-acetylpyridine adenine dinucleotide phosphate (AADP), an NADPH analog [10].

The thioredoxin system is responsible for the reduction of disulfide bonds in target proteins under physiological conditions. It is widely distributed in most types of cells, constituting one of the central antioxidants systems [1]. It is composed of a reduced substrate, a thioredoxin reductase (TR), and a thioredoxin (Trx), a conserved protein that contains an invariant WCGPC motif with the two Cys forming a redox-active intramolecular disulfide [2]. TR catalyzes the reduction of the disulfide in Trx using electrons derived from the reduced substrate, typically in the form of NAD(P)H or ferredoxin (Fdx) [3]. Thus, Trx reduces a disulfide in selected proteins via dithiol-disulfide exchange reactions, modulating the activity and/or structure of the targets and, accordingly, numerous cellular specific pathways [4,5]. Among the different types of TRs [1], Fdx-dependent flavin TRs (FFTRs) have been described in cyanobacteria and clostridia [6,7,8]. FFTRs are about 70 kDa homodimers, with each monomer comprising two domains connected by two antiparallel beta strands [6,9] (Figure 1). Each monomer contains two redox-active motifs: the FAD-binding domain, with a non-covalently bound FAD cofactor, and the redox-active disulfide domain that contains a CxxC motif, with x representing any amino acid and the two Cys forming a reversible disulfide bond. Additionally, the cyanobacterial enzyme contains a C-terminal tail with a conserved tryptophan that forms a π-stacking with the isoalloxazine ring of the opposite monomer (Figure 1). Crystal structures of the different FFTR homodimers show a central core region where the two FAD-binding domains of each protomer interact, whereas the redox-active disulfide domains display an overall open conformation that positions their CxxC motifs more than 30 Å away from the corresponding flavin [6,7,9]. Given the structural and functional homology between FFTRs and bacterial NADPH-dependent TRs (NTRs), a working model is proposed based on the flavin-reducing (FR) and flavin-oxidizing (FO) conformations adopted by bacterial NTRs during the catalytic cycle (Figure 1) [10]. Concomitant with flavin reduction, an open-to-closed conformational transition brings together the FAD and CxxC motifs for disulfide reduction (FO conformation) (Figure 1). After the intramolecular electron transfer had occurred, a closed-to-open transition (FR conformation) exposes the CxxC dithiol to the solvent for subsequent Trx reduction (Figure 1). The catalytic conformational change implies a rotation of about 66 degrees of the redox-active disulfide domain (known as NADPH-binding domain in NTRs) over the FAD-binding domain within a monomer [10]. Even though it is clear that FFTR should adopt an FO conformation during the catalytic cycle, only the FR conformation has been experimentally detected by X-ray diffraction and scattering [6,7,9].

In this study, we have used a single-molecule cutting-edge method, atomic force microscopy (AFM), to evaluate the conformational landscape of the cyanobacterial FFTR enzyme in solution, and in the presence of other components of the system. Single-molecule techniques opened up the possibility of observing individual molecules and quaternary organizations in the study of biological processes at the molecular level. These methods allow the direct sampling of distributions of molecular properties as well as the identification of different existing subpopulations, something inconceivable with bulk average ensemble biochemical and biophysical techniques [12]. Among them, AFM stands out for its potentiality and versatility, as it is the only microscopy technique that takes images of individual molecules with sub-nm resolution in near-native conditions in real-time [13].

We present the direct observation by AFM of the functional dimers from FFTR from *Gloeobacter violaceus*, GvFFTR, free and when bound to relevant partners (Trx type-m and Fdx form 1), as a tool to provide complementary insight into the FFTR catalytic cycle at pseudo-physiological conditions. GvFFTR results were evaluated in the context of equivalent data for related enzymes. These include the form 2 of FFTR from *Clostridium acetobutylicum,* CaFFTR2, and the diflavin-linked disulfide oxidoreductase from *G. violaceus*, GvDDOR. GvDDOR is also included in this study because it is structurally related to FFTR but it does not have any domain reorganization during catalysis [14] and, therefore, constitutes a good reference model for an FO conformation (Figure 1). Our results show that AFM imaging allows to morphologically differentiate between FR and FO conformations in FFTRs, as well as to topologically identify binding partners and some dynamics of the disulfide domains relative to the central core of the homodimer. Nonetheless, they evidence that under oxidizing environments, the FO state is hardly populated in GvFFTR and CaFFTR2. Therefore, to image the FO state in these TRs technical developments, allowing for AFM scanning while maintaining the FAD cofactor in the reduced state will be required in future experiments.

## 2. Materials and Methods

### 2.1. Protein Expression and Purification

Proteins used in this study (GvFFTR, GvFFTR_Δtail, GvTrxm, GvFdx1, CaFFTR2, CaTrx2, CaFFTR2:CaTrx2 covalent complex, and GvDDOR, for a complete list see Table A1) were prepared as described previously [6,7,9,14]. Briefly, proteins with an N-terminal His-tag and a Tobacco Etch Virus (TEV) protease recognition cleavage site were produced in Rosetta(DE3)pLys *E. coli* cells, and purified from the soluble extract by Ni^2+^ affinity chromatography columns. The tag was removed by a His-tagged TEV protease and further purified by a second Ni^2+^ affinity chromatography column, followed by gel filtration chromatography using a Sephacryl S-300 HR column (Cytiva) equilibrated in 20 mM Tris-HCl pH 7.6, 150 mM NaCl buffer. The covalent linked GvFFTR:GvTrxm protein complex was prepared following a similar protocol as detailed elsewhere [7] for the preparation of the CaFFTR2:CaTrx2 complex. Briefly, Cys135 and Cys35 residues of the redox active CxxC motifs in GvFFTR and GvTrxm, respectively, were changed to serine residues, resulting in GvFFTRC135S and GvTrxmC35S mutants. For the formation of the intermolecular disulfide bonds between the two proteins, Cys32 in GvTrxmC35S was conjugated to 5-thio-2-nitrobenzoic acid (TNB) that reacted with Cys138 in GvFFTRC135S and resulted in a stable covalent cross-linked complex between the two proteins, GvFFTR:GvTrxm.

### 2.2. Sample Preparation and Immobilization on Mica

AFM requires the immobilization of the biomolecules on a nano-flat surface; this ensures that they are not dragged while scanning. Protein immobilization on the mica surfaces was driven by electrostatic adsorption. Then, 0.2 µM GvFFTR, CaFFTR2, as well their variants and complexes with protein partners, and 0.4 µM GvDDOR, all in PBS pH 7.0, were incubated on freshly cleaved V-5 muscovite mica pieces (Electron Microscopy Sciences, Hatfield, UK) for 10 min at room temperature. Protein immobilization on the mica surfaces was driven by electrostatic adsorption. GvFdx1 and GvTrxm were also imaged in PBS pH 6.0 and PBS pH 7.0. Moreover, the effect of detergent molecules on the association state of wild-type flavoenzymes was checked. GvFFTR, GvDDOR, and CaFFTR2 were incubated in PBS pH 7.0 with 0.1% sodium dodecyl sulfate (SDS) and 0.2% Tween-20, and later measured in the same buffer [15]. GvFFTR and GvFFTR_Δtail were also incubated with GvFdx1 or GvTrxm proteins in a 1:2 molar ratio for 15 min under mild stirring at room temperature. Protein concentration was optimized to visualize individual separated features on further AFM images. Mica pieces were washed three times with the same buffer to release the free molecules from interfering with image acquisition, and finally covered with the same buffer ready for AFM measurements.

**Figure A1:**
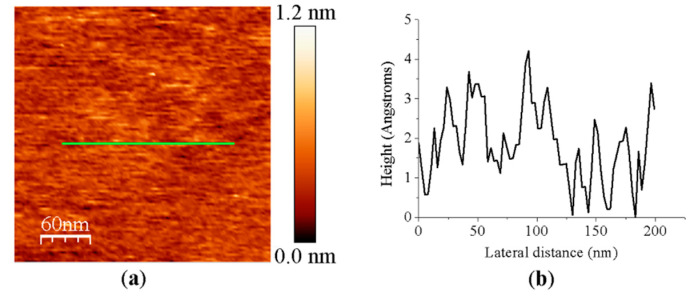
(**a**) Representative AFM image of the muscovite mica sheet surface used in this study. Scan size is 300 nm × 300 nm. (**b**) Height profile corresponding to the green line displayed on the AFM image.

To confirm that the mica background did not affect the individual experiments when imaging proteins, roughness for the bare mica surface used in this study was calculated considering the standard deviation of the Z-values of the AFM image pixels within the box cursor (Rq parameter). Five AFM images of different 300 nm × 300 nm areas were analyzed, providing an Rq parameter of 0.33 ± 0.14 nm (Appendix A Figure A1), indicating a lack of influence in the protein measurements and in consonance with previous studies.

### 2.3. Atomic Force Microscopy Imaging

AFM measurements were performed in a MultiMode 8 AFM system (Bruker, Santa Barbara, CA, USA) using the Peak Force Tapping (PFT) mode in fluid [16]. PFT mode performs a fast force curve at every pixel in the image. The peak interaction force of each force curve was used as the imaging feedback constant signal capable to adjust the relative tip-sample position and, thus, the forces applied on the soft samples. The continuous force-distance curves were acquired at a constant frequency of 1 kHz using a Peak Force amplitude of 150 nm. This operational mode is suitable to measure biological samples since very low forces—below 150 pN—are applied to the biological samples. The resolution of the images was defined as 512 × 512 pixels per area and the acquisition rate was fixed at 0.5 Hz. The above settings provided good quality images. V-shaped soft silicon nitride cantilevers exhibiting spring constants of 0.01 and 0.03 N/m (probes C and D of MSNL chips, Bruker Probes) were used. The integrated pyramidal 2 nm nominal final radius ultrasharp tips minimized the tip-sample broadening effects at the features [17]. The spring constants of the MSNL probes were calibrated by the thermal noise method [18].

The mica pieces containing the samples were introduced into an MTFML-V2 liquid cell that was filled up with the same buffer used to prepare them. At least three samples per condition were assayed. A series of topography images were collected from different areas of each sample at scan sizes ranging from 3 µm × 3 µm to 300 nm × 300 nm.

### 2.4. Image Analysis

The AFM analysis provided the direct visualization of individual features that might be attributed to enzyme molecules or their complexes with partners. At least 10 images of 10 different areas of 300–500 nm^2^ were analyzed with the WSxM free software [19]. Each feature was analyzed using the zoom and the profiles functions of the WSxM program [20]. The concentration of the enzyme incubated on the mica sheets was suitable to render isolated features identified as molecules that could be analyzed individually, allowing the conformational analysis before and upon ligand binding or in the presence of detergents. The only measurable dimension with sub-nm resolution in topology maps from AFM is Z-height, since the X and Y dimensions, which are in the plane, are somewhat enlarged by the well-known dilation tip effect [17]. Quantification of the analyzed flavoenzyme species was done as mentioned elsewhere [21].

The relative volumes of these individual features were estimated by setting a defined height threshold mask, thus eliminating any interference by background subtraction. The gathered volume data histograms were plotted to depict the mean relative volume of each species (monomer, dimer, etc.) as well as the percentage of each one of them. Frequency volume histogram plots were represented, and then treated with Gaussian functions. This strategy considers those imaged pixels of the flavoenzyme features for the given volume analysis. Populations of 100 features (*n* = 100) were analyzed for each sample condition. The raw image data were treated using Nanoscope V (Bruker, Santa Barbara, CA, USA) and WSxM [19] softwares. Estimated volumes are relative, since AFM provides greater volume values than those estimated from the crystal structures due to the tip-sample convolution effect [17], and to the swelling effect of biomolecules in liquid. Nonetheless, measuring all the samples with the same tips and in the same conditions favors the comparison of their characteristics. Furthermore, previous works reported that the molecular volumes measured with AFM compare well with the calculated volumes of the individual proteins [22]; and, in most cases, there is a clear linear correlation with the molecular weight of proteins and their complexes [23].

## 3. Results

### 3.1. AFM Allows for Comparison of Topology Patterns of FFTRs

**Figure 2:**
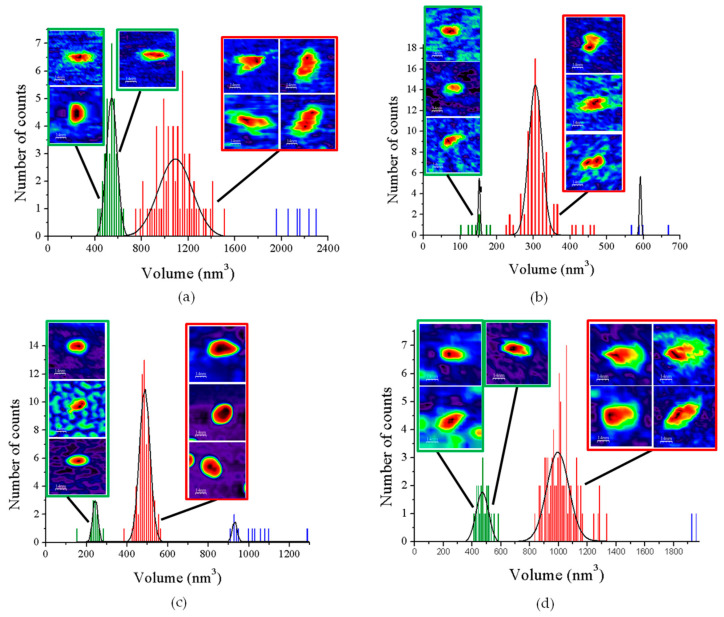
Volume frequency histograms for the features found in AFM images of native proteins. (**a**) GvFTTR; (**b**) CaFFTR2; (**c**) GvDDOR; and (**d**) GvFFTR_Δtail samples. Green, red, and blue data correspond to features assigned as subpopulations 1, 2, and 3, respectively. Besides the main subpopulations identified in each histogram, 2D images of the corresponding most representative features are shown. The sample size is defined as 100 (n = 100) for the histogram analysis. Scan sizes for all the images are 70 nm × 70 nm. The numerical ranges of “x” and “y” axes have been adjusted to the values of the histograms in each case. For clarity, the Z-scale of 2D features (differing in each case, but shown in subsequent Figures and Supplementary material Figures) and the 2D images for subpopulation 3 (which are in very small proportions) are not shown. See Figure A2 for 2D images of subpopulation 3.

**Figure 3:**
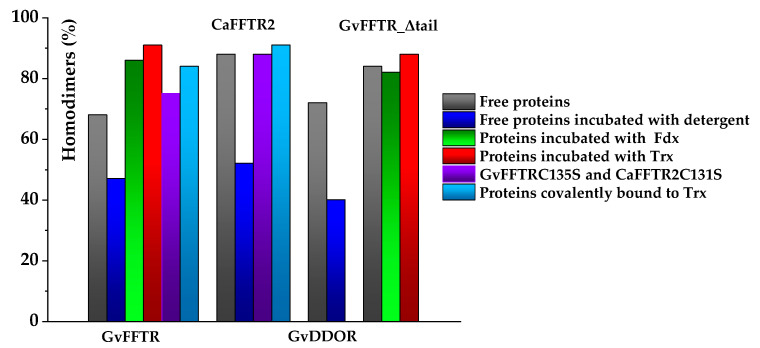
Bar chart representation of percentages of homodimers identified by AFM imaging for GvFFTR, CaFFTR2, GvDDOR, and GvFFTR_Δtail and its mixtures and covalent complexes with protein variants. Color codes for each particular condition are indicated in the right panel. Samples were measured in PBS pH 7.0. Percentages refer to the total of protomers found as homodimers of the flavoprotein (n = 100). When detergent is present, it corresponds to 0.1% SDS/0.2% Tween 20.

**Figure A2:**
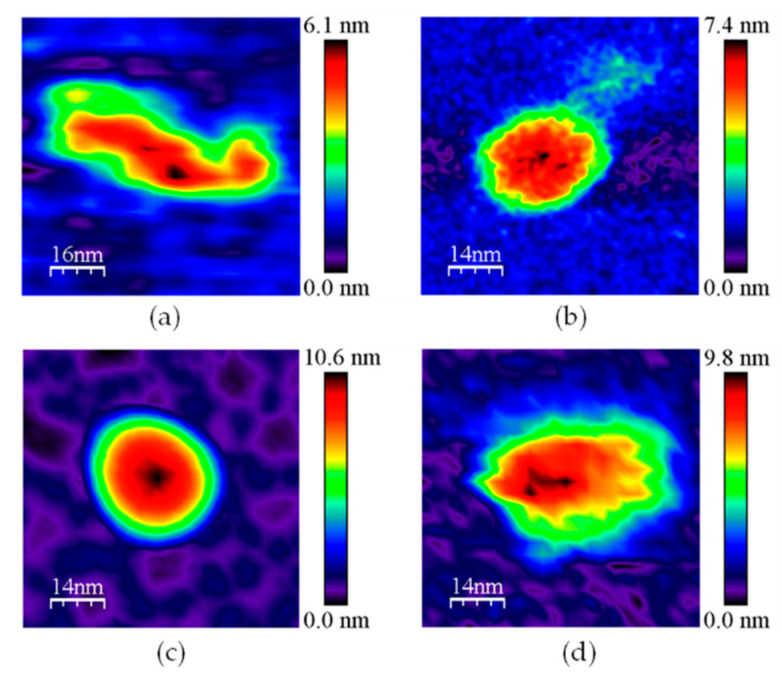
Representative 2D images for subpopulation 3 in (**a**) GvFTTR; (**b**) CaFFTR2; (**c**) GvDDOR; and (**d**) GvFFTR_Δtail samples. Samples measured in PBS pH 7.0. Scan sizes for the images are 70 nm × 70 nm, except for (**a**), 80 nm × 80 nm.

When imaging GvFFTR, CaFFTR2, GvDDOR, and GvFFTR_Δtail (a variant where the C-terminal tail has been deleted from GvFFTR, Table A1) samples on mica surfaces by AFM, we found that for all of them quantification of relative volumes was useful to identify subpopulations (Figure 2 and Figure A2, and Table A2). For these four samples, the volume frequency histograms revealed three different imaged subpopulations (Figure 2, green, red, and blue color bars for subpopulations 1, 2, and 3, respectively), whose volumes roughly duplicated regarding the previous one in each protein sample. Subpopulation 2, corresponding to intermediate volumes, resulted in a difference of being the most populated one in all samples (Figure 2). Since all these proteins are homodimers, as identified both in solution (by gel filtration chromatography and small-angle X-ray scattering measurements, SAXS) and in protein crystals [6,7,9,14], we correlated the intermediate volume subpopulation to protein homodimers (Figure 2, red bars). To confirm such assignment, GvFFTR, CaFFTR2, and GvDDOR samples were also imaged after incubation with a detergent solution formed by SDS and Tween 20 in a concentration low enough to disrupt protein–protein interactions but not to denature proteins [15]. In this way, after detergent treatment, a shift of the equilibrium towards the lower volume subpopulation was promoted (data not shown), confirming that subspecies 1 represents non-physiological protomers disrupted from homodimers (Figure 2, subpopulation 1, bars in green). Subpopulation 3 was by far the less populated and it might relate to homodimers that are imaged together or homotetramers, which are scattered and isolated (Figure 2, subpopulation 3, bars in blue; Figure A2). The observation of a small proportion of monomers under native conditions might be a side-consequence of the very low protein concentrations used for AFM imaging, far from the crowding conditions usually found in living cells. Therefore, we concluded that (as recap in Table A3 and Figure 3), under native conditions, the main features observed for GvFFTR, CaFFTR2, GvDDOR, and GvFFTR_Δtail are homodimers, which is in strong agreement with previously reported data of the physiologically functional form of these proteins [6,7,9,14].

**Figure A3:**
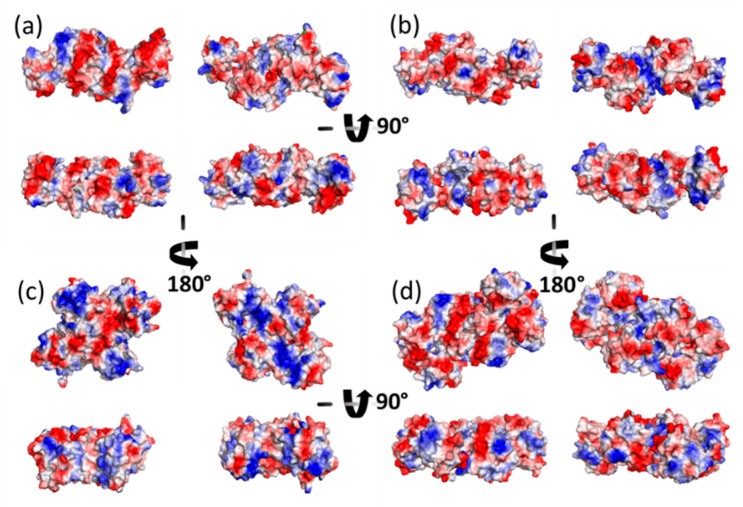
Surface electrostatic potential of homodimers for (**a**) GvFFTR, (**b**) CaFFTR2, (**c**) GvDDOR, and (**d**) the transient complex of GvFFTR with GvFdx as calculated by the PyMol Software from their PDB files (Table A1). Electrostatic potential values are shown on a scale from red through grey to blue, corresponding to −8.5, 0 and +8.5 kT/e, respectively. For each structure, the top panels represent the two 180° views of the larger lateral surfaces, while the bottom figures represent the two 180° views of their corresponding heights (bottom figures are 90° rotated with respect to the corresponding top figures). Images were produced after alignment of all structures.

Homodimers of GvFFTR, CaFFTR2, GvDDOR, and GvFFTR_Δtail have a similar number of residues (634, 582, 696, and 623 aa respectively), as well as envelope volumes estimated from 3D crystallographic structures, 98 nm^3^, 92 nm^3^, and 108 nm^3^ for GvFFTR, CaFFTR2, and GvDDOR, respectively, which were calculated using the CRYSOL Program [24]. Noticeably, estimated AFM volumes were considerably larger than those calculated from crystal structures (Figure 2, Table A2). Nonetheless, it is worth noting that the observed Z-height for dimers in the AFM images is close to the smallest dimension of the corresponding crystal structures: ~4.5, ~3.6, and ~4.0 nm at the AFM images versus ~4.3, ~3.8, and ~3.8 nm at crystal structures for GvFFTR, CaFFTR2, and GvDDOR, respectively (Figure 1 and Figure A3). Thus, in all cases, the homodimers seem to be immobilized on the mica surface by their larger dimensions (Figure 1 and Figure A3), as is often seen in AFM images [23].

AFM images are generally less precise in the X-Y dimensions, where widening tip effects increase the lateral dimensions of the biomolecules, as observed here, where enzyme molecules appear around 3–4 times larger in comparison with the crystallographic dimensions. This is manifested as an increase in the calculated volume from the images compared with the ones obtained directly from the crystal structure. However, it is worth noting the particular behavior of frequency volume histograms for GvFFTR and GvFFTR_Δtail homodimers (Figure 2)—their calculated mean volumes were nearly three and two times larger than those of the corresponding CaFFTR2 and GvDDOR homodimers, respectively.

Moreover, the envelopes of their histogram homodimer subpopulations were considerably wider than for the other two proteins (Figure 2). Such higher values and magnitude dispersion in terms of volume could be explained if, when compared with the other two proteins, GvFFTR and GvFFTR_Δtail homodimers would immobilize on the mica surface weakly and/or having a larger ability to get slightly displaced when imaged by the AFM tip. The green halo around 2D and 3D images, showing larger areas for GvFFTR and GvFFTR_Δtail when compared with the other proteins, and particularly non-existing in GvDDOR, supports this hypothesis (Figure 2). Additionally, the surface electrostatic potentials of the regions of these proteins expected to contact on the mica surface indicate that they are more negative for GvFFTRs than for the other two proteins (Figure A3). This is also in agreement with isoelectric points for GvFFTR and GvFFTR_Δtail being lower than the CaFFTR2 and GvDDOR ones (5.25 and 5.08 versus 6.53 and 6.29, respectively, as calculated from aminoacid sequences). This explains why GvFFTRs immobilize weakly and are imaged by AFM with considerably larger volumes than the other two proteins.

**Figure 4:**
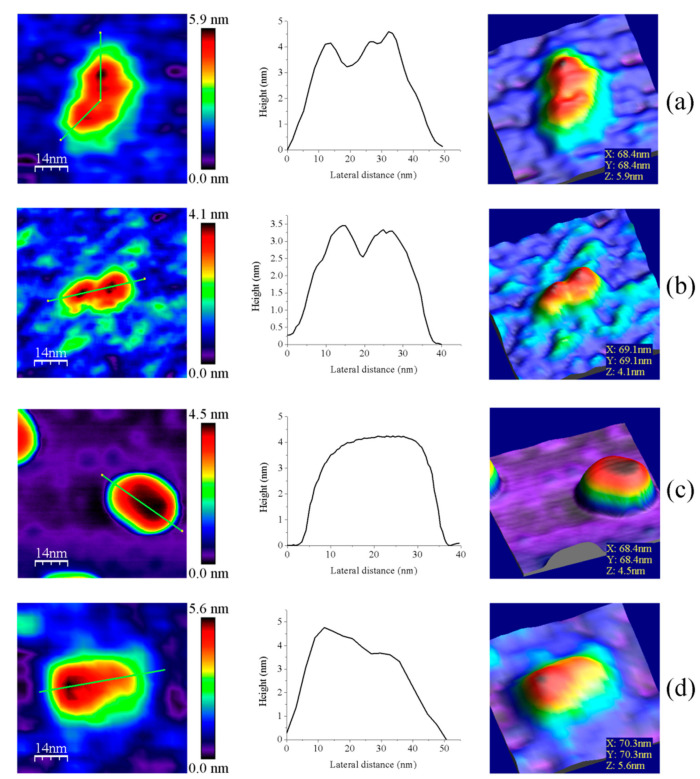
Representative images for homodimers of (**a**) GvFFTR; (**b**) CaFFTR2; (**c**) GvDDOR; and (**d**) GvFFTR_Δtail. The topography images are shown in 2D (left panels) and 3D (right panels), and the height profiles (central panels) correspond to the green lines traced on the 2D topography images. Samples were measured in PBS pH 7.0. Scan sizes for all images are 70 nm × 70 nm.

Z-height, 2D, and 3D profiles for homodimers were then carefully explored to detect potential differences in the conformation of the small redox active disulfide domain of each protomer regarding the central core formed by the FAD-binding domains of both protomers (Figure 4). GvFFTR and CaFFTR2 showed slightly higher Z-height profiles that also exhibited two peaks separated by a small valley, while a broad profile was displayed by GvDDOR. Z-height profiles for GvFFTR_Δtail also showed small changes relative to the native protein, slightly affecting the valley between peaks (Figure 4d). This later observation concurs with SAXS results which suggest that removal of the C-terminal tail slightly relaxes the conformation of the dimer [9]. Thus, the C-terminal tail is further confirmed to not significantly contribute to the overall structural conformation of GvFFTR, even though it contains the conserved tryptophan residue that stacks onto the FAD isoalloxazine ring of the adjacent protomer and is determinant for catalysis [9].

Z-height, 2D, and 3D profiles for GvFFTR and CaFFTR2 homodimers indicate that they are imaged as relatively elongated proteins (Figure 2 and Figure 4), consistent with them being in the FR basal conformation, all their redox centers being in the oxidized state, and their reported crystal structures (Figure 1 and Figure A3). In general, features appear slightly more elongated for GvFFTR than for CaFFTR2, probably due to a different relative disposition of the redox-active disulfide domain relative to its corresponding FAD-binding domain within the protomer (Figure 1 and Figure S1B from [9]). On the contrary, imaging of GvDDOR homodimers reflects a compact and rather spherical shape, in agreement with the oblate morphology found by X-ray crystallography (Figure 1 and Figure A3) for its FO conformation.

**Figure 5:**
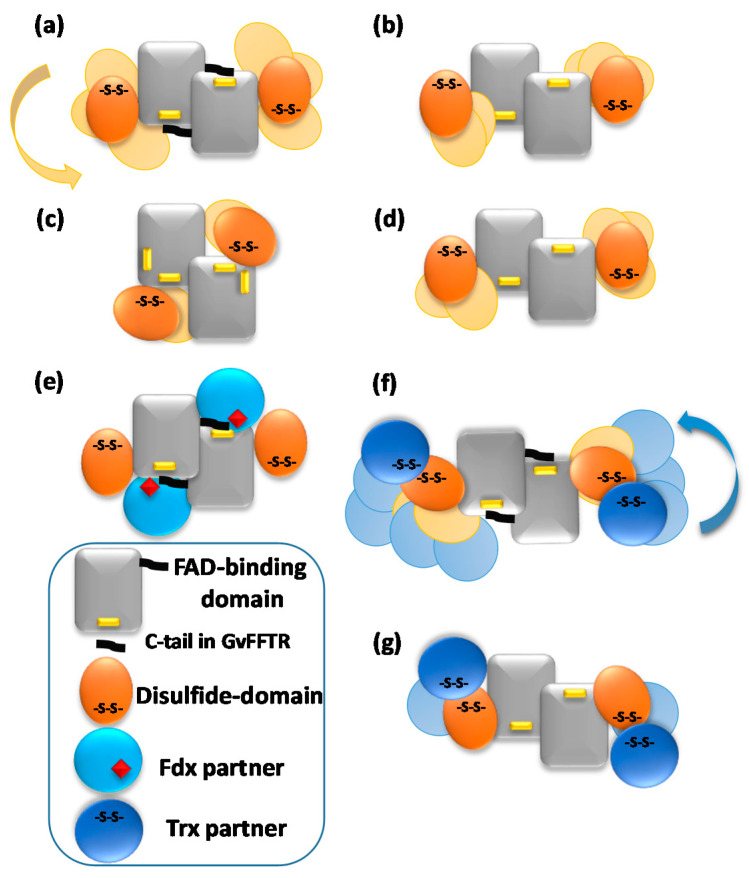
Schematic representations of variability in most representative AFM features for (**a**) GvFFTR; (**b**) CaFFTR2; (**c**) GvDDOR; and (**d**) GvFFTR_Δtail homodimers free and in mixtures of GvFFTR_Δtail and GvFdx1, as well as for (**e**) transient complexes formed in mixtures of GvFFTR and GvFdx1; (**f**) transient complexes formed in mixtures of GvFFTR and GvTrxm and covalent GvFFTR:GvTrxm and CaFFTR:CaTrx2 heterotetramer complexes; and (**g**) transient complexes formed in mixtures of GvFFTR_Δtail and GvTrxm, as well as some CaFFTR:CaTrx2 heterotetramer covalent complexes. Codes for proteins and domains are indicated at the left bottom. Solid forms represent dispositions based on crystal structures (Table A1, when available). Variability envisaged from AFM imaging of disulfide domains or protein partners regarding the central FAD-binding domains of the two protomers is shown in pale colors.

Noticeably, the 2D images recorded for GvDDOR homodimers hardly show variability, suggesting that all imaged features share a similar conformation and that it is the closed FO one (Figure 2c, see different 2D homodimer images). On the contrary, 2D images for GvFFTR and CaFFTR2 homodimers, despite displaying some particularly preferred topologies, exhibit a range of them, with some features appearing more elongated and others slightly more compact (Figure 2a,b; see 2D images for homodimers). Considering that (i) the protein-mica interactions are weaker than covalent or other protein:ligand interactions, (ii) AFM imaging enables transient dynamical conformations to occur without losing protein functionality, structure and quaternary conformations [21,25], and (iii) conformations visualized agree with other parallel single molecule studies as small angle X-ray scattering (SAXS) experiments [26] and single-molecule fluorescence resonance energy transfer (SMF-FRET) measurements [27], the AFM images here presented must reflect homodimers with a slightly different relative disposition of their disulfide domains relative to the main protein core (Figure 5). Nonetheless, all GvFFTR and GaFFTR2 images clearly reflect FR conformations, in agreement with the evaluated oxidized states.

### 3.2. Redox Protein Partners Modulate the Topology of FFTR AFM Images

**Figure 6:**
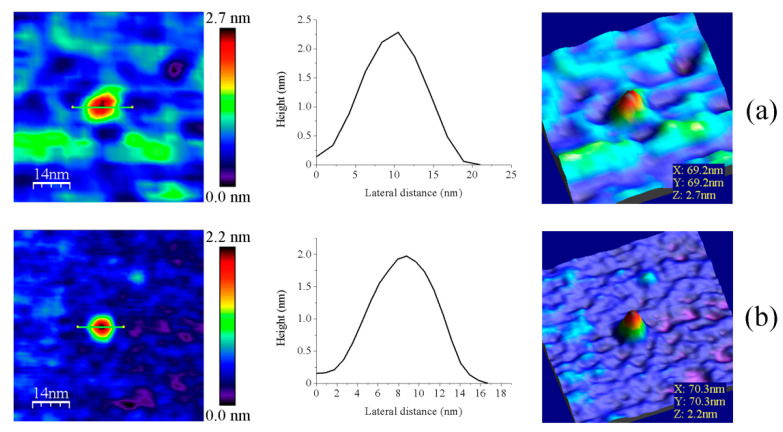
Representative main features as visualized by AFM of (**a**) GvFdx1 and (**b**) GvTrxm. Samples measured in PBS pH 6.0. Topography images are shown in 2D (left panels) and 3D (right panels), while the height profiles (central panels) correspond to the green lines traced on the 2D images. Scan sizes for all images are 70 nm × 70 nm.

Fdx and Trx proteins are, respectively, described as the physiological electron donor and receptor of FFTRs [6,9]. pH values of 6.0 were required for their immobilization on the mica surface, probably due to their lower native isoelectric points in comparison with FFTRs (the theoretical values as calculated from amino acid sequences for GvFdx1 and GvTrxm are 4.32 and 6.10, respectively). Upon AFM imaging, they showed globular features of volumes considerably smaller than those of FFTR enzymes (Figure 6, Table A2). This aspect, together with the fact that a value of ~3.9 µM is reported for the dissociation constant of the interaction of oxidized GvFdx1 with oxidized GvFFTR [9], presaged minor changes in AFM features of GvFFTR samples upon their mixing.

**Figure 7:**
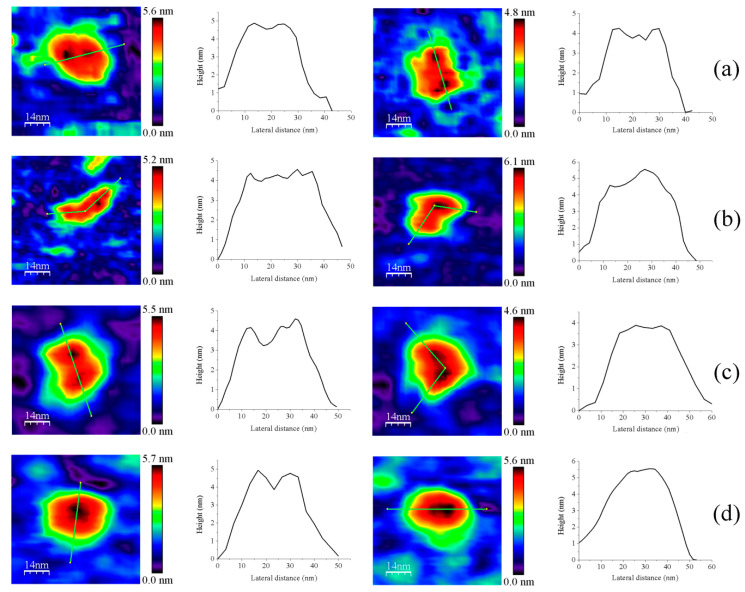
Representative main features as visualized by AFM of 1:2 mixtures of (**a**) GvFFTR with GvFdx1, GvFFTR + GvFdx1; (**b**) GvFFTR with GvTrxm, GvFFTR + GvTrxm; (**c**) GvFFTR_Δtail with GvFdx1, GvFFTR_Δtail + GvFdx1; and (**d**) GvFFTR _Δtail with GvTrxm, GvFFTR_Δtail + GvTrxm. Topography images are shown in 2D (left panels) and height profiles (on their right) correspond to the green lines traced on the 2D images. Two preferred associations are presented for each mixture. Samples were measured in PBS pH 7.0. Scan sizes for all images are 70 nm × 70 nm.

However, the subpopulation of features containing GvFFTR dimers was more abundant in GvFFTR samples mixed with protein partners; this percentage does not vary when Fdx1 is incubated with the deletion variant, but slightly increases in the presence of Trxm (Figure 3 and Figure 7, Table A3). This might be a consequence of the formation of heterotrimers/heterotetramers under some of these conditions. Moreover, the presence of the partners also modulated some Z-height profiles, which slightly increased for a number of features, as well as the topology of the 2D and 3D GvFFTR dimeric features. Thus, for samples containing GvFdx1, some of the homodimeric GvFFTR features were more spherical and compact than in free GvFFTR (Figure 7a), while minor changes were detected in the case of GvFFTR_Δtail homodimers (Figure 7c). Mixtures containing GvTrxm and GvFFTR_Δtail similarly showed features indicative of a more compact morphology than for the free enzyme (Figure 7d). On the contrary, GvTrxm promotes the appearance of a variety of morphologies and Z-heights in features representing GvFFTR homodimers, which in general are more elongated than for the free enzyme but with similar heights (Figure 7b). Thus, Z-height, 2D, and 3D profiles suggest that in some of these mixtures, together with free FFTRs, heterotrimers, and/or heterotetramers are imaged as a consequence of the interaction of Fdx1 and Trxm with the protein homodimers. This result was unexpected due to the low ratios used for the protein partners, but it might be a consequence of the repulsion of protein partners by the mica surface at pH 7.0, favoring their association to their GvFFTR receptor sites. In fact, the more spherical and compact morphology observed in GvFFTR and GvFdx1 mixtures would agree well with the surface representation of the transient complex crystal structure when compared with that of free GvFFTR (Figure 5 and compare panels (d,a) in Figure A3). This would suggest that in the imaged transient GvFFTR + GvFdx heterotrimers/heterotetramers, GvFdx1 will stack in the cliff, at the bottom of which is the FAD, filling the crevice between the FAD-binding core and the redox-active disulfide domain of one of the protomers. On the contrary, the more elongated conformations observed when GvTrxm is present, suggests that it might be binding at the apical end of protomers. This might agree with the fact that Trxm is expected to interact with the disulfide domain of one protomer in the FR conformation with minor participation of the core FAD-binding domains. This observation agrees with changes in Z-height, which are hardly being observed in this case. Noticeably, removal of the GvFFTR C-terminal tail appears to favor GvTrxm binding, contrary to that observed in the case of GvFdx1.

**Figure 8:**
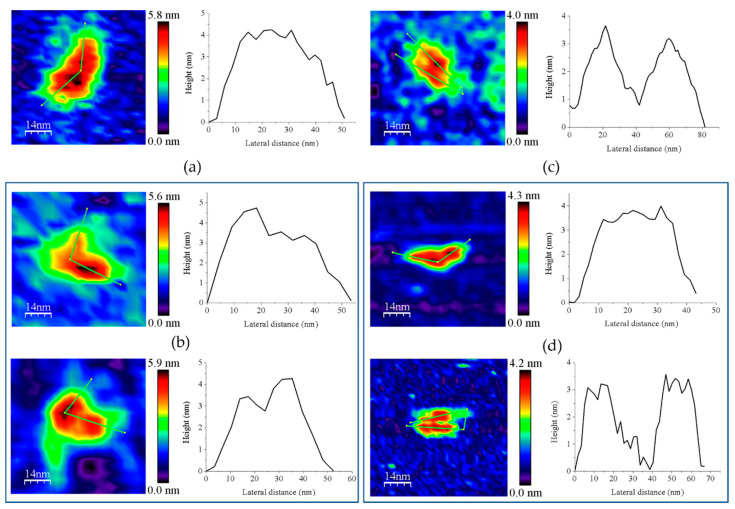
Representative main 2D features and Z-height profiles as visualized by AFM imaging for (**a**) GvFFTRC135S homodimers; (**b**) GvFFTRC135S homodimers covalently bound to GvTrxmC35S, GvFFTR:GvTrxm; (**c**) CaFFTR2C131S homodimers; and (**d**) CaFFTR2C131S homodimers covalently bound to CaTrx2C32S, CaFFTR:CaTrx2. Samples were measured in PBS pH 7.0. Z-height profiles correspond to the green lines traced on the 2D topography images. (**b**,**d**) Images for the two preferred associations observed. Scan sizes for all AFM images are 70 nm × 70 nm.

Covalent heterotetrameric complexes of GvFFTRC135S and CaFFTR2C131S variants with their corresponding GvTrxmC35S and GvTrx2C32S partners were also produced (see above) and similarly imaged to further evaluate these relevant points (Figure 8). Main AFM imaging characteristics for GvFFTRC135S and CaFFTR2C131S variants before covalent attachment to Trx proteins matched those of native proteins (Table A2, Figure 8a,c). Features for GvFFTR:GvTrxm and CaFFTR2:CaTrx2 covalent complexes in general fall within subpopulations that should be considered as heterotetramers, suggesting that the covalent binding provides a protective effect versus destabilization of FFTR homodimers in such diluted samples (Figure 3 and Table A3). Volumes for both complexes are similar to the corresponding ones for the native and mutant enzymes (Table A2), in agreement with the low size of the acceptor partner regarding the FFTR enzymes. Noticeably, topographies and Z-heights for covalent GvFFTR:GvTrxm dimers were more compact than for the relative transient interaction, with diffuse borders that indicates their relative flexibility and displacement on the mica surface. Images for CaFFTR2:CaTrx2 heterotetramers appear considerably more defined than in free CaFFTR samples, showing different degrees of elongation and slightly reducing Z-heights (Figure 8d). Thus, covalent binding of CaTrx2 to the CaFFTR homodimer produces (i) the compaction of the overall complex envelope on the mica surface and (ii) a range of different conformers representing complexes that might show a different relative disposition of the Trx2-disulfide module regarding the FAD-binding domains (Figure 8d left and right). Thus, features of CaFFTR2:CaTrx2 covalent complexes share properties in common with features of non-covalent mixtures of GvFFTR with GvTrxm (compare panels in Figure 8d with Figure 7b), suggesting that Trx binds to FFTR on the same plane it is imaged on the mica and at the apical end of the redox-active disulfide domain (Figure 5).

## 4. Discussion

AFM has been previously used to analyze protein self-association as well as protein associations with other molecules due to its capability to provide images at nm resolution in solution. The accuracy of the height profiles at sub-nm resolution and the comparison of diameters, although widened, have shown reproducible values when obtained under the same conditions. Again, the estimation of volumes based on them makes it feasible to carry out this type of analysis, and even molecular weights of yet unknown proteins have been estimated from the AFM measurement of their molecular volumes [22]. Furthermore, mica surface chemistry hardly alters the morphology, conformation, or association of the molecules, and in general, only causes the molecules, or their complexes, to be weakly immobilized by some area of their surface, preferably mainly by electrostatic adsorption through their exposed hydroxyl groups [28,29]. Therefore, imaging of enzymes by AFM at nearly physiologically relevant conditions, in the presence of reactants, products, or partners, is nowadays even used to understand catalytic pathways that occur through the formation of specific transient quaternary organizations [21,30], or even through large conformational changes upon ligand binding [26,27]. Nonetheless, there have been few studies evaluating protein morphology changes in detail [20,31,32,33,34,35,36]. In this study, the chemical modification of the mica surface to immobilize the different enzyme molecules or their complexes was not necessary, as is required for the preparation of nucleic acids and their protein complexes or acid proteins samples [37], when proteins need to be strongly attached to the surface, or even when the orientation of the molecules is crucial to be imaged or to be fish by a ligand at the AFM tip for force spectroscopy or molecular recognition experiments [38]. In the present study, we have deeply analyzed the morphologies of FFTRs, using native proteins, variants, mixtures of proteins, and covalent complex samples, to evaluate whether AFM might contribute to the topological visualization of different conformers to learn more about their catalytic mechanism. FFTRs are expected to exhibit FR and FO conformations during the catalytic cycle due to different relative dispositions between their FAD-binding and redox-active disulfide domains. However, structures have only been reported in FR conformation; hence, GvDDOR can be used as a morphological representative of the FO state.

The analysis of the AFM images of these proteins under different conditions indicates that all of them are immobilized on the mica surface, maintaining the coupling of the FAD-binding cores of the two protomers that make their physiological homodimer states. Thus, the overall immobilization and imaging procedures have a minor impact on protein integrity as previously demonstrated [21]. Our results also show that AFM imaging of these proteins can be used to distinguish between the elongated shapes of the FR states and the oblate topologies of FO states of these proteins. However, we have not been able to observe an FO state for FFTRs. Since all measurements here presented were carried out with all redox centers in oxidized state, such a result was not unexpected, and further confirms that the FO states are transient organizations happening when electron transfer from FAD to the disulfide bridge occurs. Thus, observation of FO states in FFTRs will be challenging and will require future technical developments for the in situ reduction of the FAD cofactor, while preventing electron transfer to the redox-active disulfide domain during imaging. This will be a tricky point to consider since, once the FO conformation is produced, electron transfer will occur faster than AFM imaging.

On the contrary, in NTRs, the isoalloxazine ring of the flavin cofactor is never exposed to the solvent, as they oscillate between two defined conformations that result from the rotation of the NADPH-binding domain: FO refers to the flavin cofactor close to the disulfide and FR refers to the flavin close to NADPH. Noticeably, most crystallographic structures of NTRs are found in FO conformation, independently from the redox state of either the flavin cofactor or the CxxC motif. In turn, GvFFTR contains a C-tail that stacks over the FAD of the adjacent protomer, mimicking the FR conformation both in the presence or absence of Fdx. Thereby, the switch to the FO conformation would require the C-terminal tail to flip out of the FAD. This will not be required for CaFFTR2 and GvFFTR_Δtail, because they do not have a C-terminal, preventing the access of the CxxC motif to the FAD. Nonetheless, in our experiments, we did not observed images of FO conformations for any of the evaluated TRs.

Even so, while AFM images indicate no changes in the relative disposition of the different domains in the FO conformation of the GvDDOR homodimer, they suggest that the redox-active disulfide domains of GvFFTR and CaFFTR2 can pivot slightly regarding the FAD-central core of the two protomers in the homodimer (Figure 5). When comparing these data with the two slightly different dispositions of the disulfide domains in the crystal structures for these two proteins (Figure 1), this suggests that they might represent two of the different alternate conformations they might take in solution (Figure 5). Our data also show that the formation of complexes in mixtures of FFTRs with protein partners or by covalent attachment can be detected by differences in Z-height and/or 2D and 3D profiles in AFM images. Particularly in the case of the FFTRs here evaluated, they support Trx binding at the apical end of the disulfide domain providing elongated patterns, while Fdx1 is predicted to bind closer to the protein core of the homodimer. Both observations are in agreement with the predicted mechanism for the catalytic cycle of these enzymes. Finally, the considerably large volumes calculated for GvFFTR images regarding the ones of the other two proteins indicate that the strength of interaction of the biomolecule with the mica surface will be a determinant factor to use AFM to obtain quantitative values, as the above-mentioned estimation of molecular weights.

## 5. Conclusions

In conclusion, AFM imaging appears suitable to evaluate FR and FO conformations in FFTRs and shows that under oxidizing conditions, when no electron transfer is expected, the FR conformation is the only populated species in solution. AFM also shows that in the FR conformation of FFTRs, there is a slight pivotal of the disulfide domains regarding the central homodimer, and allows to detect binding of partners by changes in Z-height, 2D, and 3D profiles. Thus far, however, imaging of FO conformations in FFTRs appears limited by the capability of being able to develop a system to keep the protein FAD cofactor reduced and the disulfide domain oxidized during AFM scanning.

## Figures and Tables

**Figure A4:**
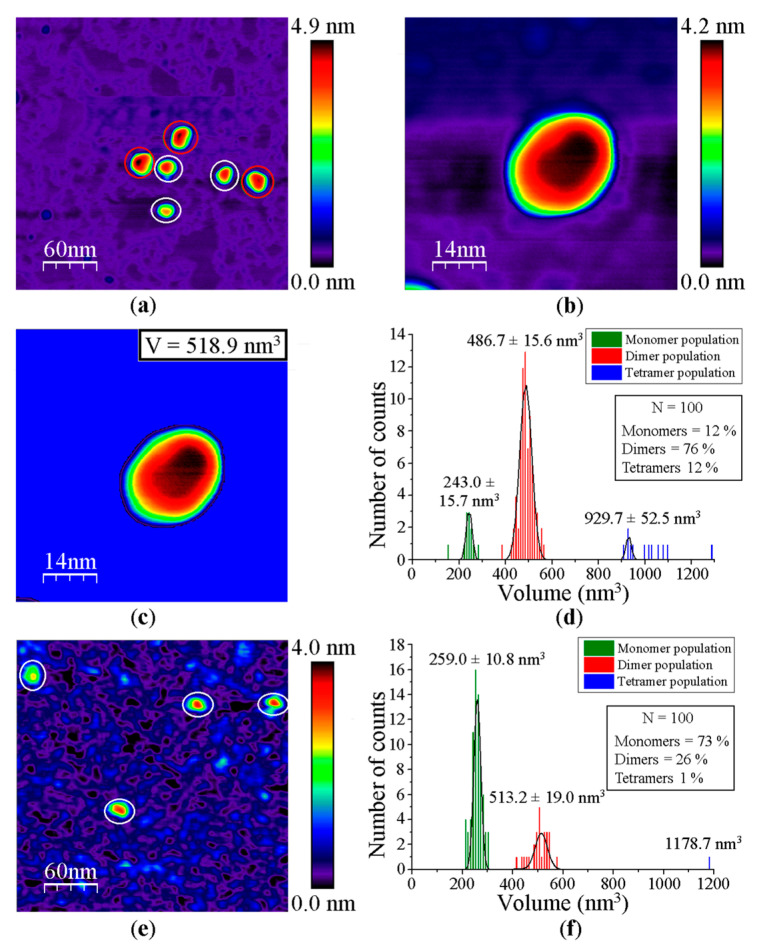
Steps for the calculation of feature volumes using as a model of study the impact of detergent molecules on the oligomerization state of GvDDOR. Data are related to GvDDOR (**a**–**d**) in the absence and (**e**,**f**) presence of detergent molecules. (**a**) AFM topography image of GvDDOR. White circles and red circles indicate monomers and dimers of GvDDOR, respectively. (**b**) AFM image of a dimer of GvDDOR. (**c**) Mask in blue to calculate the volume of the same feature depicted in (**b**). (**d**) Histogram volume analysis of the entities of GvDDOR visualized by AFM imaging. (**e**) AFM topography image of GvDDOR in presence of detergent molecules. (**f**) Histogram volume analysis of the features of GvDDOR observed in presence of detergent molecules by AFM imaging. Scan sizes are 300 nm × 300 nm for (**a**,**e**) and 70 nm × 70 nm for (**b**,**c**), respectively. n = 100 for the statistical analysis showed on (**d**,**f**). Note: White circles correspond to the green histogram distribution (GvDDOR monomers), while red circles on the AFM images are related to the red histogram distribution (GvDDOR homodimers). The choice to select white instead of red color is to optimize the contrast color for the AFM images and, thus, to make the visualization of these features easier.

## Data Availability

All primary data will be provided upon appropriate request. The data are not publicly available due to the large number of samples evaluated and images obtained.

## Supplementary Materials

The following are available online at https://www.mdpi.com/article/10.3390/antiox10091437/s1, Figure S1: Height-volume density plots for wild-type enzymes and deletion mutant, Figure S2: Height-volume density plots for protein partners and incubation mixtures, Figure S3: Height-volume density plots for FFTR variants and covalent complexes.

## Appendix A

**Table A1:** List of proteins used in this study.

	Protein	Abbreviation	Reference	PDB Code
1	Fdx-dependent flavin thioredoxin reductase from *Gloeobacter violaceus*	GvFFTR	[6]	5J60, 6XTF in complex with Fdx1
2	GvFFTR variant where the C-terminal tail, residues Ser307-His317, has been removed	GvFFTR_Δtail	[6]	n.d.
3	Thioredoxin type-m from *Gloeobacter violaceus*	GvTrxm	[6]	n.d.
4	C135S GvFFTR variant	GvFFTRC135S	This work	n.d.
5	C35S GvTrxm variant	GvTrxC35S	This work	n.d.
6	Covalent complex of C135S GvFFTR and C35S GvTrx	GvFFTR:GvTrxm	This work	n.d.
7	Ferredoxin 1 from *Gloeobacter violaceus*	GvFdx1	[9]	6XTF in complex with GvFFTR
8	Diflavin-linked disulfide oxidoreductase from *Gloeobacter violaceus*	GvDDOR	[14]	5ODE
9	Fdx-dependent flavin thioredoxin reductase 2 from *Clostridium acetobutylicum*	CaFFTR2	[7]	6GNC
10	Thioredoxin 2 from *Clostridium acetobutylicum*	CaTrx2	[7]	6G9N
11	C131S CaFFTR2 variant	CaFFTR2C131S	[7]	n.d.
12	C32S CaTrx2 variant	CaTrx2C32S	[7]	n.d.
13	Covalent complex of C131SCaFFTR2 and C32SCaTrx2	CaFFTR2:CaTrx2	[7]	6GND

**Table A2:** Estimated relative volumes (mean ± SD) for the features corresponding to the different protein samples analyzed in this work. Samples were measured in PBS pH 7.0, except for GvFdx1 and GvTrxm, which were analyzed at PBS pH 6.0. * Volume values showing statistically significant differences from the corresponding free GvFFTR, GvFFTR_Δtail, or CaFFTR2 homodimers, as determined by the Student’s test (*p* < 0.05).

Sample	Subpopulation 1	Subpopulation 2	Subpopulation 3
	Monomers (nm^3^)	Homodimers ^a^ (nm^3^)	Homotetramers (nm^3^)
GvFFTR	544 ± 25	1080 ± 82	2133 ± 62
CaFFTR2	151 ± 13	305 ± 20	590 ± 20
GvDDOR	243 ± 16	487 ± 16	930 ± 52
GvFFTR_Δtail	474 ± 22	1003 ± 52	1942 ± 20
GvFdx1	63 ± 3		
GvFFTR + GvFdx1	508 ± 21	1120 ± 64 *	2071
GvFFTR_Δtail + GvFdx1	501 ± 20	1023 ± 68 *	2113
GvTrxm	41 ± 2		
GvFFTR + GvTrxm	498 ± 13	1069 ± 58 *	
GvFFTR_Δtail + GvTrxm	507 ± 15	1048 ± 55 *	
GvFFTRC135S	505 ± 21	1020 ± 65	2121 ± 114
GvFFTR:GvTrxm	494 ± 14	1053 ± 55 *	2211 ± 78
CaFFTR2C131S	157 ± 8	313 ± 22	643
CaFFTR2:CaTrx2	158 ± 8	309 ± 19 *	

^a^ This subpopulation is considered to contain FFTR and DDOR homodimers, as well as their complexes with protein partners. These later organizations will be, therefore, heterotrimers and/or heterotetramers.

**Table A3:** Distribution of subpopulations identified by AFM for GvFFTR, CaFFTR2, GvDDOR, GvFFTR_Δtail, GvFdx1, and GvTrxm, as well as of mixtures of FFTRs with partners (+) and when covalently attached to them (:). Samples imaged in PBS pH 7.0. Percentages refer to the total of protomers found in each association state (n = 100). ^#^ Stands for distributions in the presence of 0.1% SDS/0.2% Tween 20 detergents. n.d., not determined.

Sample	Monomers (%)	Homodimers ^a^ (%)	Homotetramers (%)
GvFFTR	19/53 ^#^	68/47 ^#^	13/n.d. ^#^
CaFFTR2	4/48 ^#^	88/52 ^#^	8/n.d. ^#^
GvDDOR	6/57 ^#^	72/40 ^#^	22/3 ^#^
GvFFTR_Δtail	12	84	4
GvFFTR + GvFdx1	11	86	3
GvFFTR_Δtail + GvFdx1	15	82	3
GvFFTR + GvTrxm	8	91	n.d.
GvFFTR_Δtail + GvTrxm	12	88	n.d.
GvFFTRC135S	21	75	4
GvFFTR:GvTrxm	10	84	6
CaFFTR2C131S	10	88	2
CaFFTR2:CaTrx2	9	91	n.d.

^a^ This subpopulation is considered to contain FFTR and DDOR homodimers, as well as their complexes with protein partners, potential heterotrimers, and/or heterotetramers.
