# Supplementary material for: Atomic Force Microscopy to Elicit Conformational Transitions of Ferredoxin-Dependent Flavin Thioredoxin Reductases
Source: Antioxidants (Basel). 2021 Sep 9;10(9):1437. doi: 10.3390/antiox10091437 (PMC8469568; doi:10.3390/antiox10091437)
Supplement: Supplementary file 1 [file antioxidants-10-01437-s001.zip › antioxidants-1338586-supplementary.pdf]

SUPPLEMENTARY MATERIALS

Atomic Force Microscopy to elicit conformational transitions of ferredoxin-dependent flavin thioredoxin reductases.

Carlos Marcuello [1,4], Gifty Animwaa Frempong [1], Mónica Balsera [2], Milagros Medina [3] and Anabel Lostao [1,4,5]

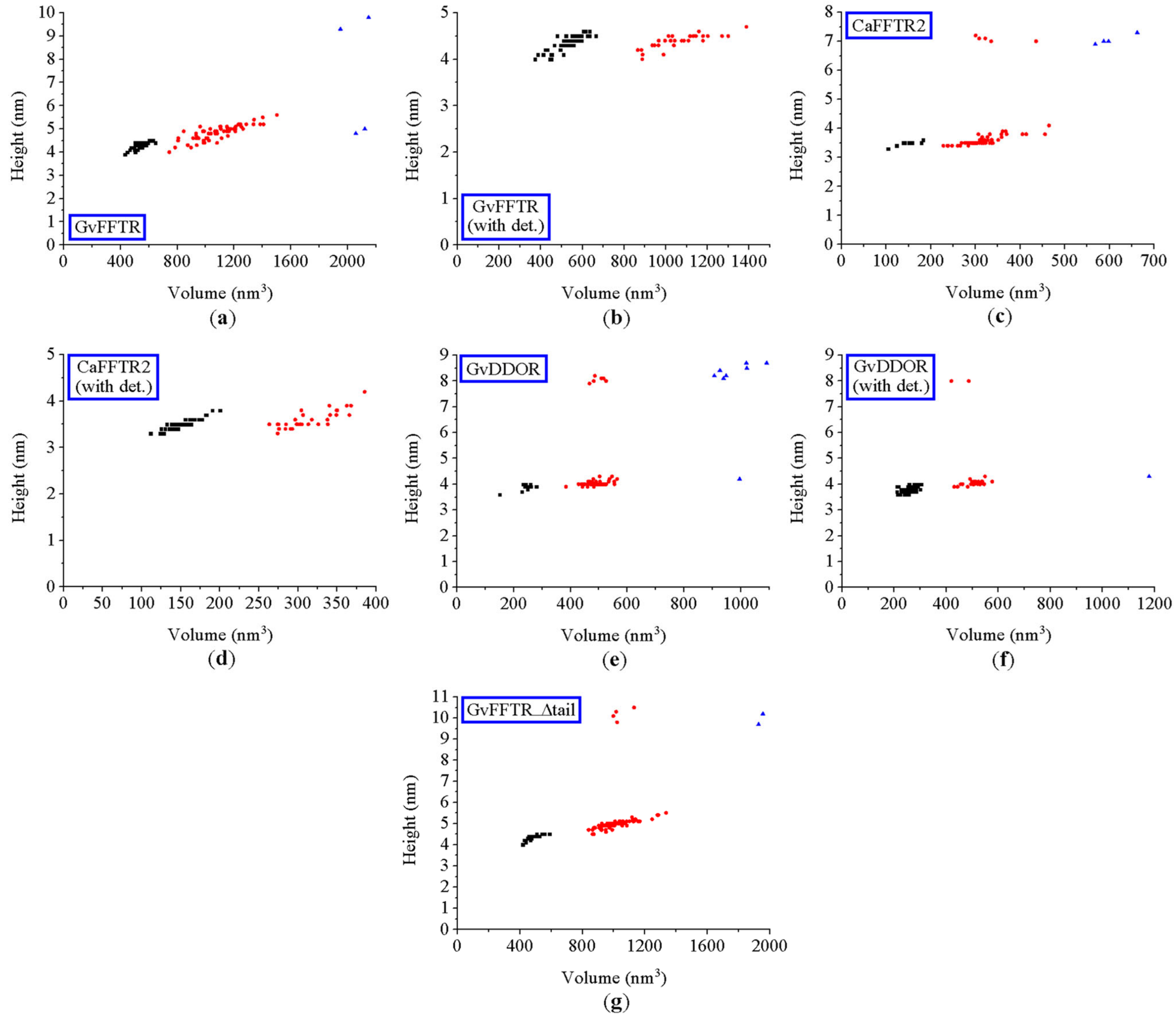

Figure S1. Height-volume density plots for wild-type enzymes and deletion mutant. (**a**) GvFFTR; (**b**) GvFFTR with detergents; (**c**) CaFFTR2; (**d**) CaFFTR2 with detergents; (**e**) GvDDOR; (**f**) GvDDOR with detergents; and (**g**) GvFFTR_Δtail. Black squares, red circles and blue triangles correspond to monomer, dimer and tetramer protein subpopulations, respectively. N = 100 for all cases.

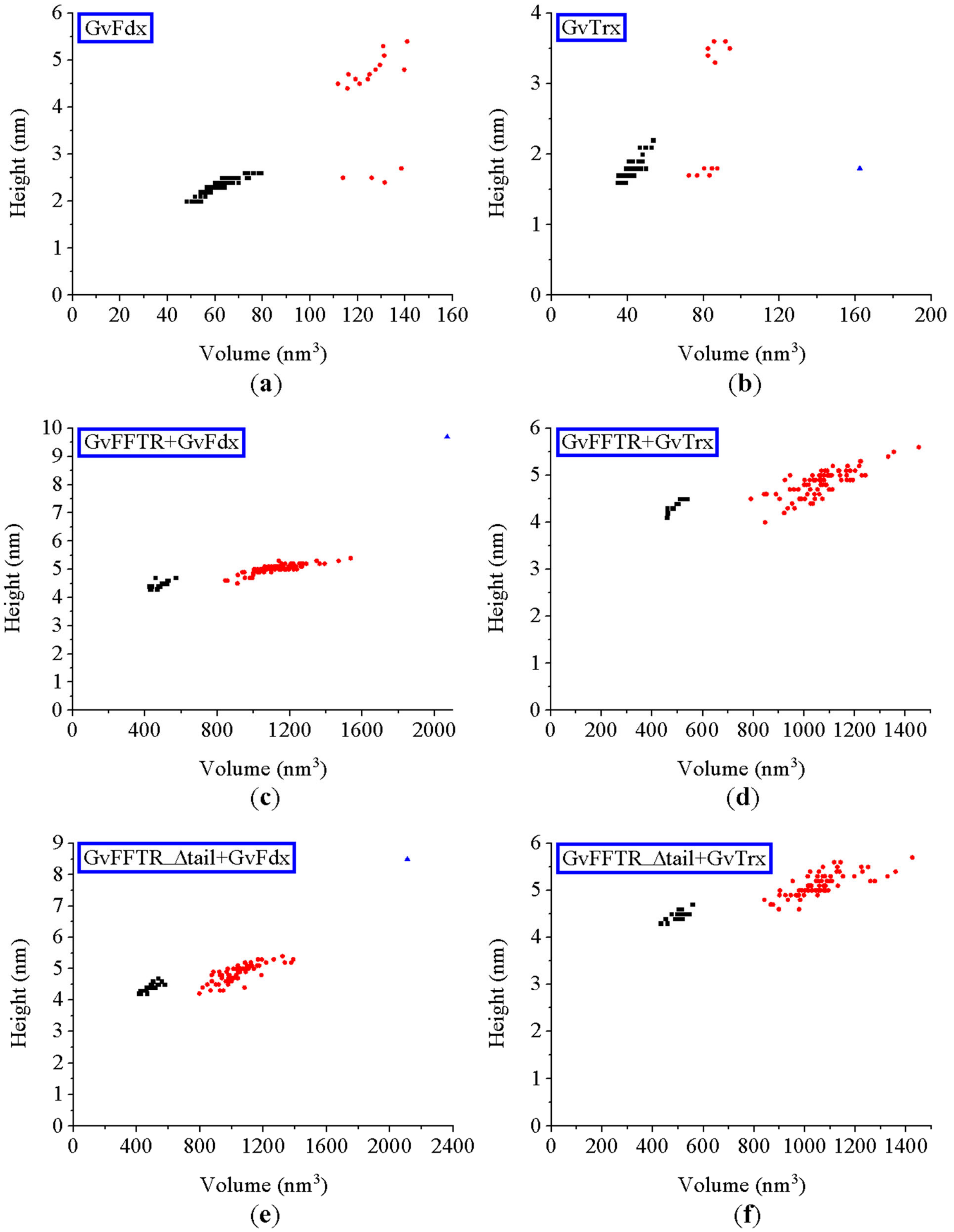

Figure S2. Height-volume density plots for protein partners and incubation mixtures. (**a**) GvFdx; (**b**) GvTrx; (**c**) GvFFTR with GvFdx, GvFFTR+GvFdx; (**d**) GvFFTR with GvTrx, GvFFTR+GvTrx; (**e**) GvFFTR_Δtail with GvFdx, GvFFTR_Δtail+GvFdx and (**f**) GvFFTR_Δtail with GvTrx, GvFFTR_Δtail+GvTrx. Black squares, red circles and blue triangles correspond to monomer, dimer and tetramer protein subpopulations, respectively. N = 100 for all cases.

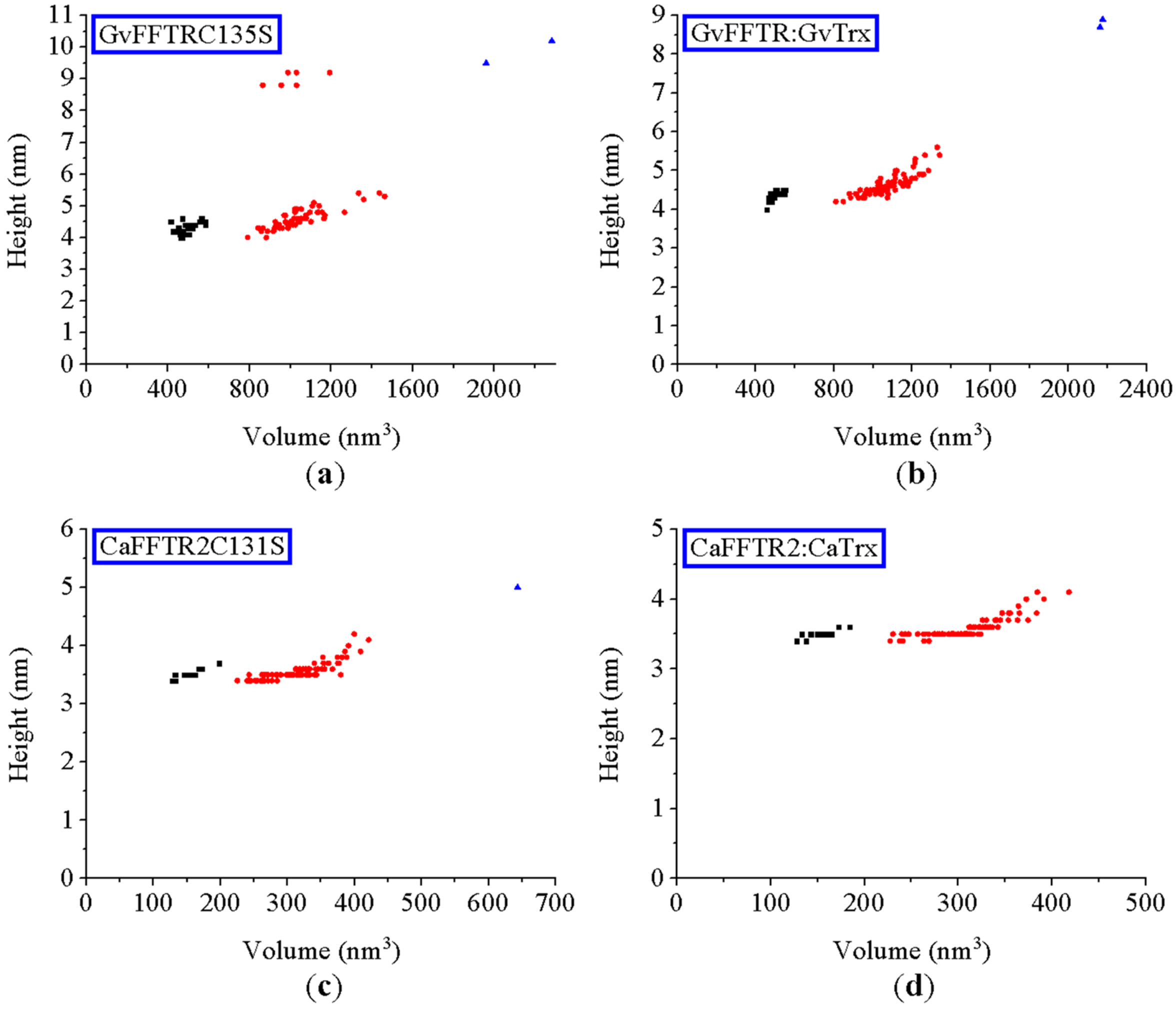

Figure S3. Height-volume density plots for FFTR variants and covalent complexes. (**a**) GvFFTRC135S; (**b**) GvFFTRC135S covalently bound to GvTrxm, GvFFTR2:GvTrx; (**c**) CaFFTR2C131S and (**d**) CaFFTR2C131S covalently bound to CaTrx, CaFFTR:2:CaTrx. Black squares, red circles and blue triangles correspond to monomer, dimer and tetramer protein subpopulations, respectively. N = 100 for all cases.
